# Ontogenetic regulation of metabolite dynamics in *Thymbra capitata*: temporal reconfiguration of essential oils and antioxidant capacities across sequential harvests

**DOI:** 10.7717/peerj.21519

**Published:** 2026-07-08

**Authors:** Uğur Tan

**Affiliations:** 1 Department of Field Crops, Faculty of Agriculture, Aydın Adnan Menderes University, Aydın, Turkey; 2 Integrated Molecular Plant Physiology Research, Department of Biology, Universiteit Antwerpen, Antwerpen, Belgium

**Keywords:** Developmental stage, Sequential harvests, Metabolite trade-off, Antioxidant activity, Carvacrol

## Abstract

**Background:**

*Thymbra capitata* is a Mediterranean aromatic shrub that is extensively used to produce essential oils and phenolic antioxidants. Despite having a chemical profile that varies with phenological stage, most previous studies have been based on single or few harvests, giving only a fixed perspective of metabolism. As a result, the ontogenetic control of essential oil production, monoterpene formation, and antioxidant-related metabolism throughout the entire vegetation cycle has been poorly clarified. The aim of this study was to measure such changes in these characteristics over sequential periods of harvest to determine developmental trade-offs between essential oils and antioxidants under natural conditions.

**Methods:**

Plant samples were collected bi-weekly (H1–H14) from late winter through late summer. Essential oils were extracted by hydrodistillation and their composition analyzed by gas chromatography–mass spectrometry (GC-MS). The Folin-Ciocalteu, Aluminium chloride colorimetric, 2,2-diphenyl-1-picrylhydrazyl (DPPH), and 2,2′-azinobis(3-ethylbenzothiazoline-6-sulfonic acid) (ABTS) assays were used to measure total phenolic content (TPC), total flavonoid content (TFC) and antioxidant activities. Mixed-effects models with unequal variance structures were employed in the analysis of data, and principal component analysis was used to examine multivariate patterns in essential oil composition.

**Results:**

The content of essential oils showed high ontogenetic modulation, increasing from 1.38% at the initial harvest to a maximum of 5.97% at flowering (H11) with a 333% increase, and followed by a 46% decrease during post-flowering senescence. The highest levels of TFC were reached in initial harvest as 225.83 mg rutin g^−1^ dry weight (DW) at H2 and decreased by 49% at the final harvest. The TPC ranged between 41.58 mg gallic acid equivalents (GAE) g^−1^ DW and 65.47 mg GAE g^−1^ DW, with the highest value occurring in the middle of the season. DPPH activity reached 64.47 mg Trolox equivalent antioxidant capacity (TEAC) g^−1^ DW at H5 and dropped to a minimum value at H13–H14. The essential oil composition also changed with time, with carvacrol decreased from 81.2% at H1 to 64.6% at H14, and p-cymene (cymol) and γ-terpinene increased from 2.6% to 13.4% and 3.8% to 9.3% respectively.

**Conclusions:**

These findings demonstrate that phenolic-related antioxidants and terpenoid volatiles in *T. capitata* undergo coordinated, developmentally driven changes that result in a quantitative trade-off between these metabolite groups. Early harvest maximizes the levels of antioxidants and phenolic monoterpenes, whereas flowering and late harvests favor higher essential oil with a hydrocarbon-rich profile. Understanding these temporal metabolic patterns provides a practical background for optimizing harvest timing to achieve specific industrial, nutritional, or therapeutic objectives.

## Introduction

Aromatic and medicinal plants biosynthesize a large variety of secondary metabolites that have important ecological roles and high value for humans (Wang et al., 2024). Among these metabolites, essential oils are the most remarkable as complex mixtures of volatile terpenoids responsible for characteristic aromas and bioactivities of herbs in the family Lamiaceae (Abdelmohsen & Elmaidomy, 2025; Yu et al., 2025). In the genus *Thymus* and some other genera and species, essential oils may also reach up to ∼90% monoterpenes in content, often with a higher predominant phenolic content (thymol and carvacrol) accompanied by biogenetic precursors (p-cymene and γ-terpinene) (Casiglia et al., 2019). These constituents form strong antimicrobial and antioxidant properties, which are the basis of the traditional use of thyme herbs for natural preservatives and remedies (Trong & Thinh, 2023).

*Thymbra capitata* is also referred to under the synonyms of *Thymus capitatus* L. and *Coridothymus capitatus* is one of the remarkable examples of a Mediterranean perennial shrub belonging to Lamiaceae family and is important in the culinary and ethnomedical fields (Bruno, Badalamenti & Cerasola, 2025; Nehme et al., 2025). Commonly known as Spanish oregano or conehead thyme, it grows in xerophilous soils in the Mediterranean basin, and it is especially common around the coastal areas of southern Iberia (Arantes, Caldeira & Martins, 2022; Nehme et al., 2025). In northern Morocco, it is called locally “Zaïtra” where its leaves are used extensively as a culinary herb as well as a medicinal remedy (El Hadj Ali, Guetat & Boussaid, 2012). Traditional herbalists and current ethnopharmacological works acknowledge the plant for its wound healing, anti-inflammatory, and respiratory benefits (Alves-Silva et al., 2023; Rijo, 2024), but the molecular mechanisms of these properties remain poorly understood. Essential oils distilled from *T. capitata* are rich in phenolic monoterpenes particularly carvacrol, thymol, p-cymene and γ-terpinene which confer broad antimicrobial, antioxidant and antifungal activities, making the species valuable to the food, cosmetic and pharmaceutical industries (Basım, 2025; Bruno, Badalamenti & Cerasola, 2025).

Essential oils of this species are typically dominated by phenolic monoterpenes, particularly carvacrol, often exceeding 60–80% of the total composition, although thymol-dominant chemotypes have also been reported in certain regions (Blanco Salas et al., 2010; Bakhy et al., 2013; Russo et al., 2013). For example, comparative surveys across the Mediterranean reveal substantial chemotypic variation; for example, one study found carvacrol and γ-terpinene co-dominant in Ramallah, Palestine (central), γ-terpinene paired with cis-β-terpineol in Jenin, Palestine (north), and thymol with β-caryophyllene in Hebron, Palestine (south) (Jaradat et al., 2025). It is also reported in other studies there are significant intraspecific variability in *Thymbra capitata* essential oils, with distinct chemotypes characterized by thymol, carvacrol, or mixed profiles (Miceli, Negro & Tommasi, 2006; Al Hafi et al., 2017; Hanoglu et al., 2017). Such spatial heterogeneity is attributed to genetic differentiation and environmental gradients such as altitude, rainfall, and soil type, yet a shared feature across studies remains the predominance of monoterpene hydrocarbons and their oxygenated derivatives (Pirbalouti et al., 2025).

In addition to essential oil constituents, *T. capitata* is also rich in phenolic compounds such as rosmarinic acid and flavonoids, which contribute significantly to its antioxidant capacity (Saija et al., 2016). The biological activities of its essential oil, including antioxidant, antimicrobial, and anti-inflammatory effects, are largely attributed to this phenolic-rich composition (Carrasco et al., 2016; Goudjil et al., 2020; Zaïri et al., 2019). Furthermore, the major compounds of *T. capitata* essential oil, including carvacrol and thymol, are derived from a common biosynthetic pathway involving precursors such as p-cymene and γ-terpinene (Skoula & Grayer, 2005).

Despite extensive research on *T. capitata*, most studies have focused on essential-oil composition during a single phenological stage usually flowering or have compared only a few harvest times (*e.g.*, pre- *vs.* post-flowering in one season, or regional chemotype differences at one time) (Casiglia et al., 2019; Moukhles et al., 2019). This restricts our understanding of the dynamic changes in both essential and non-essential phytochemical throughout plant development. In this context, a time-resolved sequential harvesting approach was implemented to investigate the influence of developmental stages on essential oil yield and composition, as well as phenolic and flavonoid contents and antioxidant activity. This comprehensive arrangement enables simultaneous assessment of essential oil yield and composition, phenylpropanoid-derived metabolites (total phenolics and flavonoids), and antioxidant capacity, alongside dynamic changes in monoterpene biosynthetic pathways.

## Materials & Methods

### Plant material and climate

**Figure 1:**
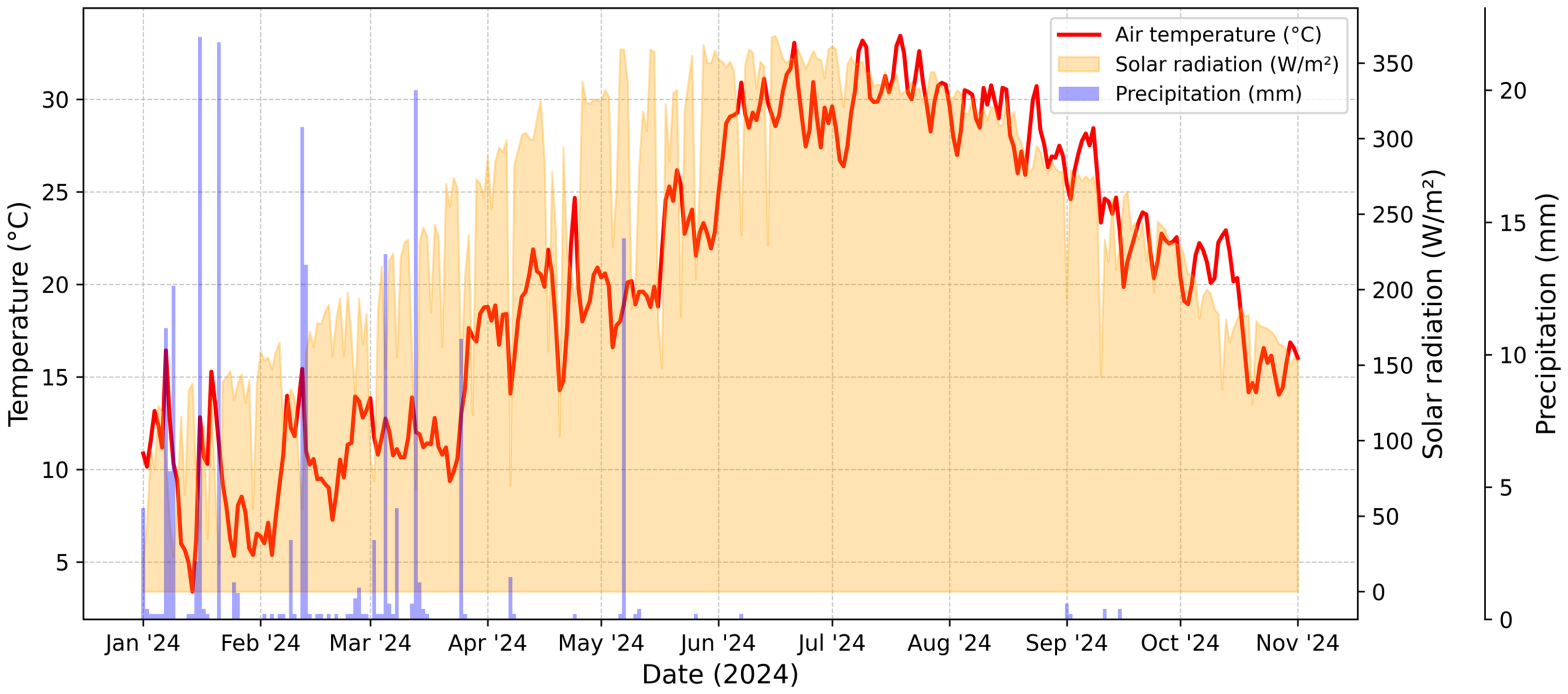
Temporal variation in climatic parameters during experimental period (Jan–Nov 2024). Air temperature (°C), solar radiation (W m^2^), and precipitation (mm) are presented as a function of time.

Naturally growing *Thymbra capitata* plants were used as the plant material. The author was responsible for the species identification. Plant samples were gathered every two weeks from three separate sampling sites identified within the Faculty of Agriculture at Aydin Adnan Menderes University, located in the Koçarli, Aydin region. No field permits were required for this study, as the work was conducted in an accessible area and did not involve protected species, private property, or restricted sites. The climatic data monitored during the experimental period shows the existence of distinct seasonal dynamics in the study area (Fig. 1). The air temperature gradually rose throughout the early part of the year, reaching its peak in midsummer before declining through autumn. Solar radiation followed a similar seasonal pattern, with higher levels during the summer months associated with longer day lengths. However, precipitation’s distribution over time remains inconsistent, with events happening sporadically rather than following a regular seasonal curve.

### Experimental design and sample preparation

The study was designed using three spatially separated sampling sites established within a single study area (approximately 300 m^2^). Each site represented one biological replicate (4 m^2^), resulting in a total of three biological replicates per sampling date. Samples were collected biweekly from each site. The experiment ran from February 27, 2024, to August 28, 2024, until the vegetation cycle concluded, meaning the plants were mostly dried. Prior to analysis, the collected plant material was allowed to dry for one week in the shade at room temperature (approximately 22–24 degrees Celsius). After the drying period, the samples were separated from stems, leaves, and flowers manually. The stems were discarded, and only the combined leaf and flower fraction was used for analysis, using two technical replicates to ensure the accuracy of the analyses. Leaves and flowers were not analyzed separately because the main goal of this study was to characterize the general above-ground phytochemical profile rather than variation between organs.

### Essential oil content (%)

The essential oil content (EOC) was quantified volumetrically from dried leaves using a Neo-Clevenger apparatus, with yields expressed as mL per 100 g of air-dried material. The procedure was carried out based on the method of Wichtl (1971), with minor modifications. A 10-gram sample of plant material underwent hydrodistillation for 1 h, after procedure, the volume of collected essential oil in Neo-Clevenger was measured. The measured value was then calculated as a percentage according to the formula given below.

\begin{eqnarray*}Essential~Oil~Content~(\%)= \frac{Volume~of~Essential~Oil~Obtained~(mL)}{Dry~Sample~Weight~(g)}  \times 100. \end{eqnarray*}

### Essential oil component analysis

Essential oil components were analyzed using a Shimadzu GCMS-QP2010 SE gas chromatography–mass spectrometry system (Shimadzu Corporation, Kyoto, Japan) equipped with an Rxi-5Sil MS capillary column (30 m × 0.25 mm i.d. × 0.25 µm film thickness; Restek Corporation, Bellefonte, PA, USA). Helium (99.999%) was used as the carrier gas at a constant flow rate of 1.00 mL min^−^^1^. Samples were injected in split mode (1:10) with an injector temperature of 280 °C. The oven temperature was initially held at 60 °C. The column flow, total flow, and linear velocity were 1.00 mL min^−^^1^, 14.0 mL min^−^^1^, and 36.5 cm s^−^^1^, respectively. The system pressure was 57.4 kPa and the purge flow was 3.0 mL min^−^^1^. The mass spectrometer was operated in electron ionization (EI) mode. The ion source and interface temperatures were set at 250 °C and 280 °C, respectively. Compound identification was performed by comparing mass spectra with those in the Wiley, NIST, and FFNSC libraries. Retention indices (RI) were calculated relative to a homologous series of n-alkanes (C7–C30) analyzed under identical chromatographic conditions. The calculated RI values were compared with literature data for confirmation. Compound identification was accepted based on agreement between mass spectral data and retention indices with those reported in the literature. For each harvest stage, one representative sample was analyzed by GC–MS.

### Sample preparation and extraction

Dried plant samples were ground with a grinder and passed through a sieve in order to obtain uniformity for the extraction procedure. For the extraction, 500 mg of the powdered samples were mixed with 50 ml of 80% methanol in a shaking incubator at 40 °C for 2 h. After extraction, 20 ml of the extract was dried in an oven at 70 °C until constant weight was achieved, and the yield of the extract was calculated on a dry weight (DW) basis (Maisuthisakul, Suttajit & Pongsawatmanit, 2007). The extracts were filtered using Whatman no. 42 filter paper. The filtered samples were used for determination of total phenolic content (TPC), total flavonoid content (TFC), 2,2-diphenyl-1-picrylhydrazyl (DPPH) and 2,2′-azinobis (3-ethylbenzothiazoline-6-sulfonic acid) (ABTS) values.

### DPPH radical scavenging assay

The antioxidant analysis was performed using the DPPH assay with slight modifications, following the method described by Gadow, Joubert & Hansmann (1997) and Maisuthisakul, Suttajit & Pongsawatmanit (2007). A 100 µL extract was added to 3,900 µL of freshly prepared DPPH solution. Freshly prepared samples were 0.15 mM in MeOH. The extract was used directly as obtained from the extraction process without any further dilution. The mixture was shaken and incubated in the dark at room temperature for 30 min. The final solution’s absorbance was measured spectrophotometrically at 516 nm using a microplate reader (Multiskan GO; Thermo Fisher Scientific, Vantaa, Finland). The DPPH values of the samples expressed in terms of Trolox equivalent antioxidant capacity (mg TEAC g^−1^ DW).

### ABTS•^+^ radical scavenging assay

The ABTS assay was performed as described by Re et al. (1999). To form the ABTS radical cation (ABTS•+), a solution of 7 mM ABTS was mixed with 2.45 mM potassium persulphate (1:1 ratio) and allowed to stand in the dark for 16 h. The resulting solution of (ABTS•+), was then diluted with methanol to provide 0.700 absorbance at 734 nm. An aliquot of 5 ul of plant extract was added to 3.995 mL diluted ABTS•+ solution and incubated for 30 min in the dark. After that, the absorbance of the sample was measured, and the antioxidant capacity was expressed as TEAC equivalent (mg TEAC g^−1^ DW). A microplate reader (Multiskan GO; Thermo Fisher Scientific, Vantaa, Finland) was used for measurements.

### Total phenolic content analysis

TPC was determined using the Folin–Ciocalteu method (Skerget et al., 2005). To perform this procedure, a mixture of a sample extract 0.5 mL, 2.5 mL of 0.1N Folin-Ciocalteu’s reagent, and two mL of sodium carbonate solution (75 g L^−1^ Na_2_CO_3_) is prepared. The reaction mixture was then incubated at 50 °C for 5 min to maintain the formation of the blue colour, which was an indicator of the presence of the phenolic chemicals. After incubation, the mixture was shocked on ice and returned to room temperature. The absorbance of the solution was then measured at 760 nm with a microplate reader (Multiskan GO; Thermo Fisher Scientific, Vantaa, Finland). TPC was represented in gallic acid equivalents (mg GAE g^−1^ DW).

### Total flavonoid content analysis

TFC was determined using the aluminium chloride colorimetric method according to Chang et al. (2006). To quantify the TFC, a 0.5 mL aliquot of the sample extract was combined with 2.5 mL of distilled water and 150 microliters of a 5% sodium nitrite solution. This mixture was then carefully mixed. The mixture was allowed to stand for 5 min, then 300 µL of aluminum chloride (AlCl_3_) 10% was added and allowed to react for another 5 min. Then, one mL of 1 M sodium hydroxide (NaOH) was added, and the volume was completed with distilled water to five mL. The solution was then incubated for 30 min, and the absorbance was detected at 510 nm with a microplate reader (Multiskan GO; Thermo Fisher Scientific, Vantaa, Finland). The contents of flavonoids were expressed as Rutin trihydrate (mg rutin g^−1^ DW) equivalent (MW: 664.56) according to the method of Chang et al. (2006).

### Statistical analyses

Before conducting the analysis, the assumptions of analysis of variance (ANOVA) were checked, and Levene’s test revealed that the variances across the groups were not uniform. A mixed-effects model accounting for heteroscedasticity was implemented, incorporating an unequal variance structure. The sampling site was included as a random effect to account for repeated measurements over time. *Post hoc* pairwise comparisons were performed using Tukey-adjusted least-squares means, which provide valid multiple-comparison inference under unequal variances. All statistical analyses were conducted in JMP Pro 18 (SAS Institute, Cary, NC, USA), while data visualization using Python’s Matplotlib library and PCA analyses were conducted using Python within the JupyterLab environment.

## Results

### Essential oil content, phenolic and flavonoid contents, and antioxidant activity

**Figure 2:**
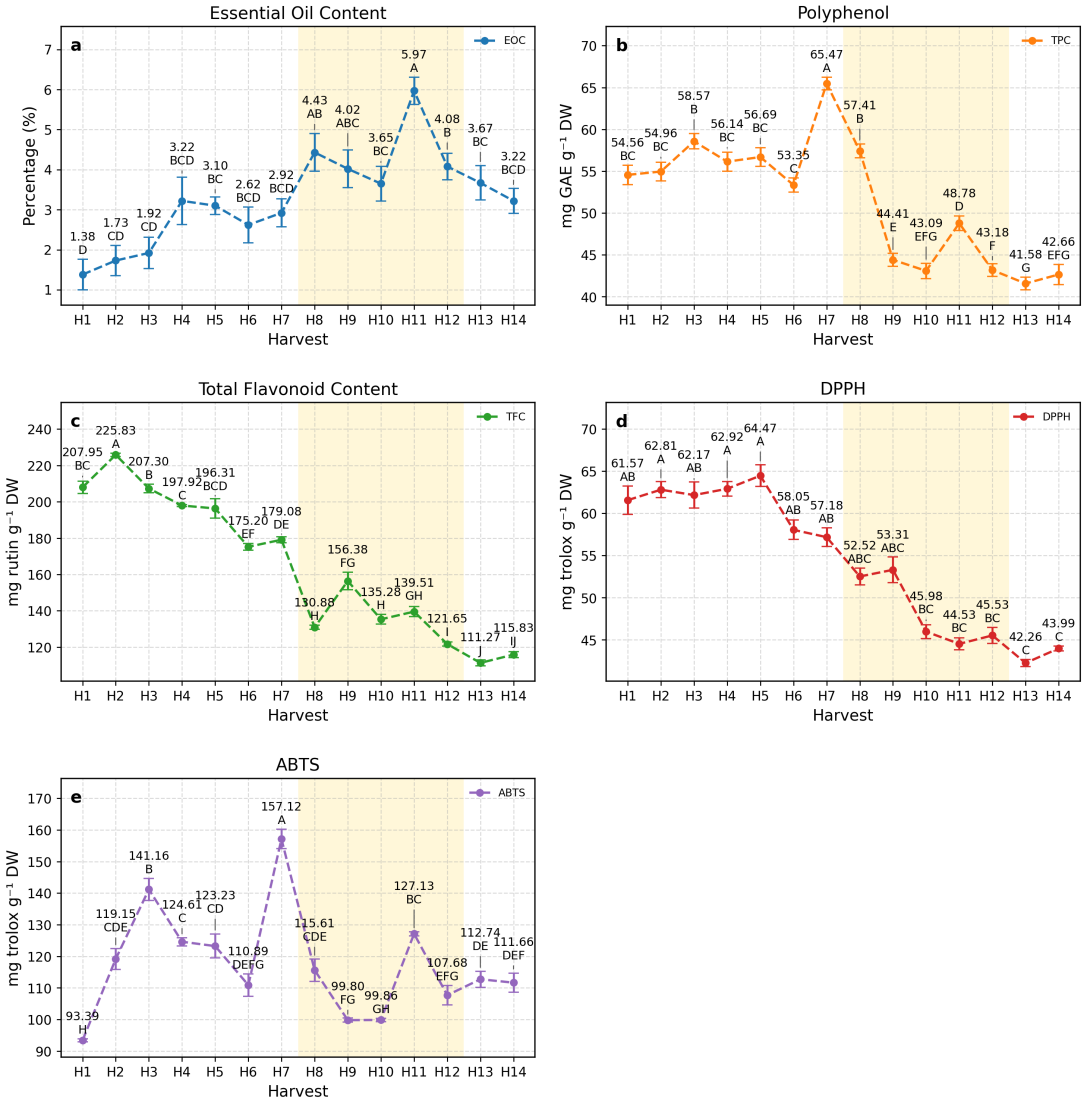
Temporal variation in essential oil content, phenolic and flavonoid levels, and antioxidant activity across fourteen harvest stages (H1–H14). Yellow shaded area indicates the flowering period. (A) Essential oil content (EOC, %). (B) Total phenolic content (TPC, mg GAE g^−1^ DW). (C) Total flavonoid content (TFC, mg rutin equivalents g^−1^ DW). (D) DPPH radical scavenging activity (mg TEAC g^−1^ DW). (E) ABTS radical scavenging activity (mg TEAC g^−1^ DW).

The quantitative patterns found during the fourteen harvests indicate clear temporal changes in the accumulation of essential oils and antioxidant capacity (Fig. 2). EOC showed the most drastic variation: the EOC increased from 1.38% at the first harvest to 5.97% at H11 during flowering, representing a 333% increase, and thereafter declined by 46% to 3.22% at H14 during post-flowering. TFC also exhibited huge fluctuations, reaching the maximum value of 225.83 mg g^−1^at H2 and decreasing by about 49% at the last harvest. Antioxidant activities showed similar trends but of different orders of magnitude: ABTS radical scavenging capacity increased by ∼68% between H1 and its mid-season maximum at H7, followed by a decrease of ∼29% at the end of the season, DPPH values increased slightly early in the season, reaching a maximum of 64.47 mg g^−1^ at H5, followed by a decrease of ∼1/3 at H14. TPC was 57% higher from the lowest (H13) to the highest (H7) and then declined by ∼35% towards the last harvest. These percentage changes indicate that the harvest time can cause dramatic changes both in magnitude and in the distribution of phytochemical traits.

### Changes in essential oil composition across sequential harvests

**Figure 3:**
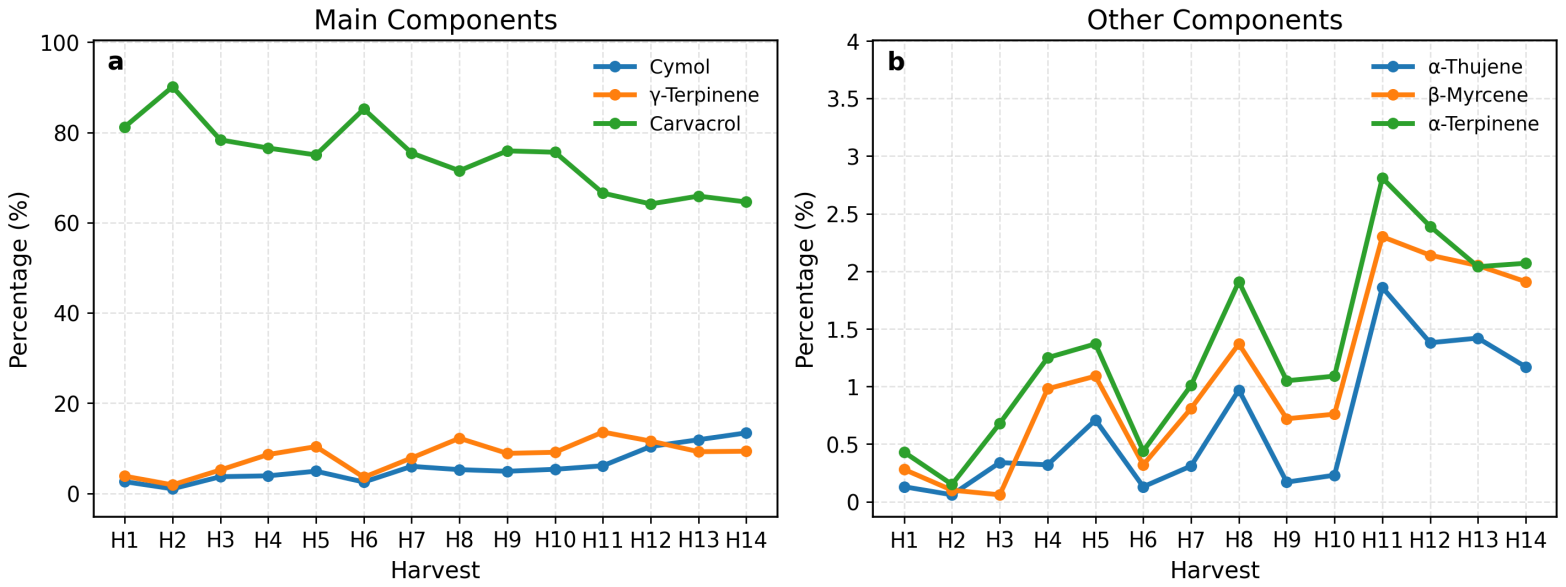
Harvest-dependent variation in essential oil composition across fourteen sequential harvests (H1–H14). (A) Relative percentages of major components (carvacrol, p-cymene, and γ-terpinene). (B) Relative percentages of selected minor components (α-thujene, β-myrcene, and α-terpinene).

The series of fourteen sequential harvests (H1-H14) showed clear changes in the essential oil composition, with the monoterpenoids p-cymene (cymol), γ-terpinene and carvacrol being accountable for the most part of the observed variability (Fig. 3). At the first harvest, carvacrol was predominant (about 81% of the total) while p-cymene and γ-terpinene were found in low quantities (2.6% and 3.8%). Over time, the levels of the latter two compounds increased to 13.4% and 9.3%, respectively, by the last harvest, whereas carvacrol decreased to approximately 64.6%. Several minor constituents showed disproportionate increases even at low absolute levels. α-Thujene increased from 0.13% at H1 to 1.86% at H11, β-myrcene from 0.28% to 2.30% and α-terpinene from 0.43% to 2.81% over the same period. Although these hydrocarbons never approached the abundance of the major compounds, their enrichment is an indication of an intensification of the monoterpene hydrocarbon fraction. By grouping structural classes, it is possible to note that monoterpene hydrocarbons increased from 7.53% to 30.44% between H1 and H14, while oxygenated monoterpenes decreased from 90.32% to 67.95% (Table 1). Most other constituents, pinene isomers, sabinene, linalool, borneol, terpinen-4-ol and sesquiterpenes were below 1% and were largely background. The high identification percentage (around 99–100%) in all the harvests indicates the completeness of the compositional analysis.

Within oxygen-containing monoterpenes, linalool, borneol, and terpinen-4-ol were consistently present, but their relative contribution was small compared with carvacrol and variable throughout the series (Table 1). Linalool was in the range of 0.48% (H11) to 2.75% (H4) and was relatively more in the early to mid-harvest (*e.g.*, 2.24−2.75% in H3–H4) and less in the latter harvest (0.65−0.86% in H12–H14). Borneol showed a strong decrease throughout the sequence with high early concentrations (3.34% in H1; 2.51% in H3) and lower concentrations in the later harvests (0.47% in H11; 0.94−1.20% in H12–H14). Terpinen-4-ol varied between 0.31% (H4) to 1.04% (H10) although it was around 0.6−1.0% in most harvests, and slightly higher in H7–H10 and H14. trans-Sabinene hydrate displayed a progressive decrease in the dataset, from 0.87% in H3 and 0.79% in H1 to 0.25% in H14, suggesting that this oxygenated monoterpene was more characteristic of early-season vegetative regrowth.

**Figure 4:**
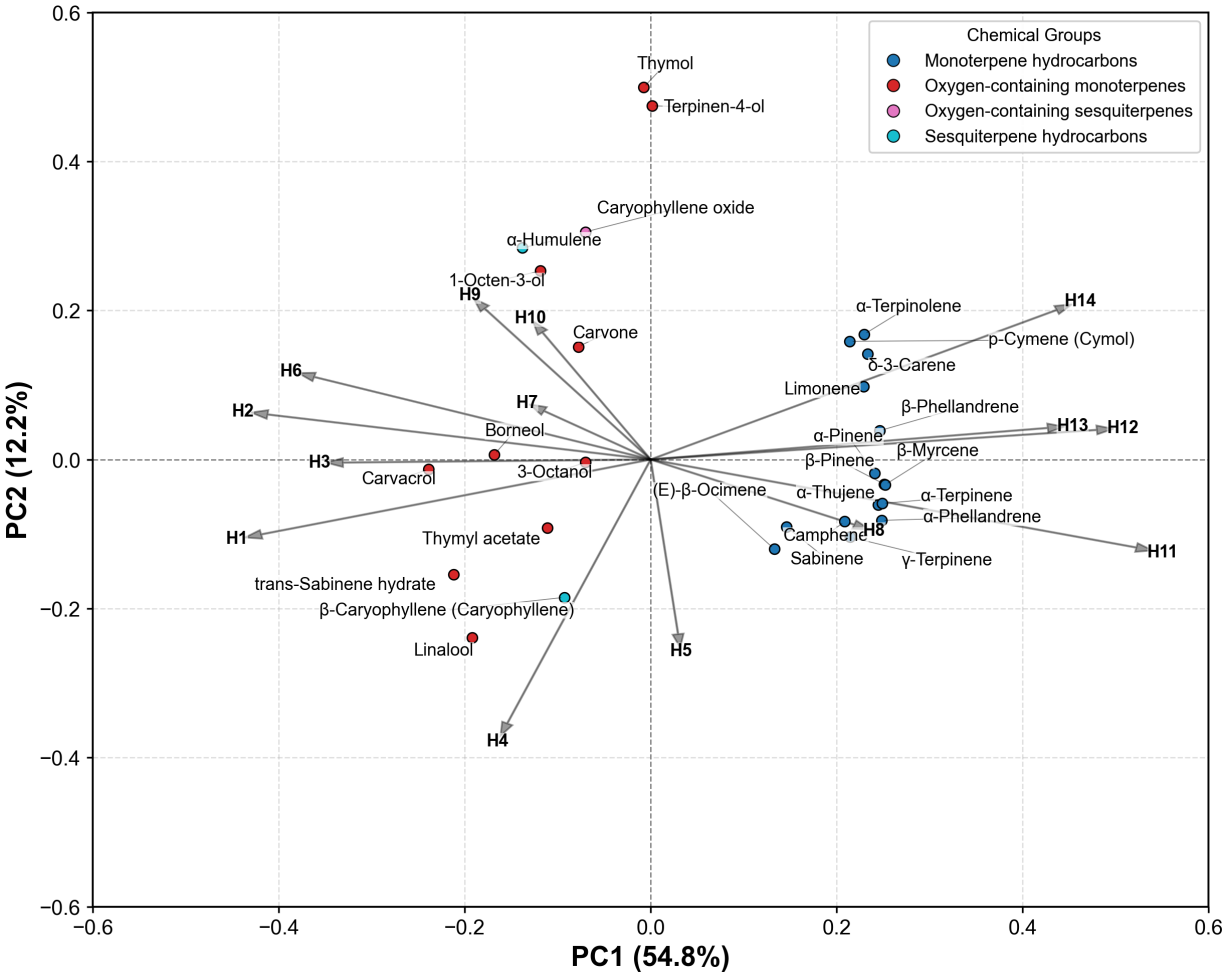
Principal component analysis (PCA) biplot of essential oil components of *Thymbra capitata* across fourteen sequential harvest stages (H1–H14). Points represent individual compounds grouped by chemical class, while vectors indicate the association of harvest stages with compositional variation.

A temporal segregation of harvest stages based on changes in EOC was identified in the PCA biplot. Early harvests (H1–H4) are concentrated on the left side of PC1 and are correlated with oxygenated monoterpenes represented by trans-sabinene hydrate, linalool, borneol, thymol acetate, and carvacrol (Fig. 4). Mid-season samples (H5–H9) are in the middle and are associated with mixed profiles with both oxygenated and hydrocarbon monoterpenes. Late-season harvests (H10–H14) are strongly shifted to the right along PC1 because of high levels of hydrocarbon monoterpenes such as a-thujene, v-myrcene, a-pinene, limonene, g-terpinene, and p-cymene. Overall, PC1 (54.8%) describes the shift from oxygenated monoterpenes being dominant in the early growth stages to hydrocarbon monoterpenes being dominant in later stages, and PC2 (12.2%) describes secondary differences between specific oxygenated compounds. The distinct gradient from H1 to H14 suggests a progressive, developmentally driven reorganization of terpenoid metabolism throughout the harvest sequence.

**Table 1:** Relative composition (%) of essential oil components of *Thymbra capitata* across fourteen sequential harvests (H1–H14), determined by GC–MS.

**Components**	**RT**	**RI** ^**a**^	**RI** ^**b**^	**H1**	**H2**	**H3**	**H4**	**H5**	**H6**	**H7**	**H8**	**H9**	**H10**	**H11**	**H12**	**H13**	**H14**
α-Thujene	6.25	923	924	0.13	–	0.34	0.32	0.71	0.13	0.31	0.97	0.17	0.23	1.86	1.38	1.42	1.17
α-Pinene	6.502	930	932	0.09	–	0.25	0.22	0.36	0.08	0.18	0.46	0.09	0.11	0.77	0.75	0.96	0.77
Camphene	7.064	946	946	0.1	–	0.22	0.19	0.16	0.05	0.1	0.28	0.05	0.06	0.22	0.36	0.4	0.31
Sabinene	7.888	970	969	–	–	–	–	–	–	–	0.05	–	0.01	0.04	0.03	–	–
β-Pinene	8.088	976	974	–	–	0.02	0.07	0.08	0.02	0.05	0.12	0.03	0.04	0.21	0.2	0.2	0.17
1-Octen-3-ol	8.194	979	979	0.26	0.21	0.41	0.1	0.04	0.19	0.71	0.19	0.44	0.44	0.06	0.15	0.06	0.14
β-Myrcene	8.524	987	988	0.28	0.1	0.57	0.98	1.09	0.32	0.81	1.37	0.72	0.76	2.3	2.14	2.05	1.91
3-Octanol	8.915	997	995	–	–	0.07	–	–	–	–	–	–	–	–	–	–	–
α-Phellandrene	9.251	1,005	1,002	0.03	–	0.07	0.13	0.17	0.03	0.12	0.2	0.09	0.1	0.35	0.28	0.25	0.23
*δ*-3-Carene	9.373	1,008	1,008	–	–	–	–	–	–	0.06	0.06	0.03	0.03	0.11	0.1	0.12	0.1
α-Terpinene	9.734	1,015	1,014	0.43	0.15	0.68	1.25	1.37	0.44	1.01	1.91	1.05	1.09	2.81	2.39	2.04	2.07
p-Cymene (Cymol)	10.089	1,024	1,026	2.61	1.02	3.71	3.9	4.93	2.52	6	5.27	4.91	5.35	6.13	10.4	11.9	13.42
Limonene	10.305	1,027	1,030	0.03	0.04	0.27	0.17	0.19	0.12	0.21	0.25	0.18	0.2	0.36	0.42	0.45	0.45
β-Phellandrene	10.364	1,028	1,032	–	–	–	0.08	0.18	–	0.1	0.18	0.13	0.11	0.29	0.28	0.22	0.33
1,8-Cineole	10.425	1,029	1,033	–	0.29	0.27	–	0.05	0.13	0.19	0.06	0.16	0.15	0.04	0.04	–	–
(E)-β-Ocimene	11.172	1,043	1,044	–	–	0.03	–	0.08	–	–	0.04	0.04	–	0.06	0.06	–	0.03
γ-Terpinene	11.78	1,055	1,059	3.83	1.9	5.2	8.62	10.38	3.58	7.86	12.23	8.88	9.12	13.6	11.6	9.25	9.35
trans-Sabinene hydrate	12.355	1,070	1,070	0.79	0.78	0.87	0.72	0.8	0.69	0.71	0.56	0.75	0.71	0.57	0.49	0.38	0.25
α-Terpinolene	13.167	1,085	1,086	–	–	–	–	0.05	–	0.04	0.07	0.09	0.08	0.14	0.14	0.1	0.13
Linalool	14.016	1,100	1,095	1.85	1.6	2.24	2.75	1.49	1.37	1.79	0.94	1.2	1.18	0.48	0.65	0.69	0.86
Borneol	18.175	1,168	1,165	3.34	1.05	2.51	1.56	0.68	1.6	1.41	1.24	1.61	1.68	0.47	1.2	1	0.94
Terpinen-4-ol	18.736	1,177	1,174	0.72	0.73	0.65	0.31	0.37	0.61	0.9	0.63	0.92	1.04	0.63	0.75	0.62	0.92
Carvone	23.439	1,260	1,243	–	–	0.06	–	–	–	–	–	0.07	–	–	–	–	–
Thymol	26.134	1,307	1,290	0.21	0.37	0.27	0.14	0.2	0.45	0.29	0.25	0.42	0.41	0.26	0.35	0.31	0.39
Carvacrol	26.965	1,317	1,298	81.22	90.11	78.35	76.54	75.01	85.19	75.44	71.52	75.91	75.61	66.56	64.15	65.89	64.59
Thymyl acetate	30.761	1,374	1,352	2.19	–	0.9	–	–	–	–	–	–	–	–	–	–	–
β-Caryophyllene	33.913	1,412	1,418	1.48	1.06	1.43	1.95	1.42	1.77	1.32	0.98	1.7	1.24	1.43	1.31	1.32	1.04
α-Humulene	36.221	1,449	1,454	–	0.02	0.04	–	–	0.04	–	–	0.06	0.03	–	–	–	–
Caryophyllene oxide	43.849	1,573	1,582	0.41	0.36	0.37	–	0.19	0.62	0.23	0.17	0.25	0.19	0.09	0.3	0.35	0.43
*% Identification*				100	99.79	99.8	100	100	99.95	99.84	100	99.95	99.97	99.84	99.92	99.98	100
*Grouped components*																	
Monoterpene hydrocarbons				7.53	3.21	11.36	15.94	19.75	7.29	16.85	23.46	16.46	17.29	29.25	30.53	29.36	30.44
Oxygen-containing monoterpenes				90.32	94.93	86.12	82.02	78.6	90.04	80.73	75.21	81.04	80.78	69.01	67.63	68.89	67.95
Sesquiterpene hydrocarbons				1.48	1.08	1.47	1.95	1.45	1.81	1.32	0.98	1.76	1.27	1.43	1.31	1.32	1.05
Oxygen-containing sesquiterpenes				0.41	0.36	0.37	0	0.19	0.62	0.23	0.17	0.25	0.19	0.09	0.3	0.35	0.43
Phenylpropanoids				–	–	–	–	–	–	–	–	–	–	–	–	–	–
Others				0.26	0.21	0.48	0.1	0.04	0.19	0.71	0.19	0.44	0.44	0.06	0.15	0.06	0.14

**Notes.**

RT Retention times

RI^a^ : Retention indices (RI) were determined experimentally on an Rxi-5Sil MS capillary column relative to a homologous series of n-alkanes (C7–C30) under the same chromatographic conditions.

RI^b^ : Literature retention index values were obtained from the NIST and used for comparison. H1–H14 represent sequential harvest times conducted at biweekly intervals. Representative GC–MS chromatograms of the first (H1) and last harvest (H14) are provided in the Supplementary Material (Figs. S1 and S2). The composition of each compound is expressed as a percentage of the total identified components.

## Discussion

### Ontogenetic trade-offs in phenolics and essential oils

Previous studies on *Thymbra capitata* have consistently reported that essential oil yield is strongly influenced by phenological stage, with maximum production typically occurring during the flowering period (Hedhili et al., 2002; Moukhles et al., 2019). Our results are in strong agreement with these findings, as EOC increased progressively and reached its peak at the flowering stage (H11). However, unlike previous studies that were limited to a few sampling points, our high-resolution sequential sampling reveals the full temporal trajectory of this increase and the subsequent decline during post-flowering senescence. The temporal profiles of the measured phytochemicals indicate a phase-dependent reconfiguration of secondary metabolism. Essential oil concentration was more than tri-fold greater between the first harvest (H1) and peak flowering (H11) followed by a >50% decline over senescence.

These trends seem likely to be a change in metabolic flow between the phenylpropanoid and terpenoid pathways as the plant develops (Liu et al., 2024; Petrović et al., 2024). Phenylpropanoid metabolism originates from phenylalanine *via* phenylalanine ammonia lyase and chalcone synthases, which divert carbon to phenolic acids and flavonoids (Ninkuu et al., 2025). The hydroxylated structures of these compounds give them strong radical scavenging properties, and their accumulation is tightly regulated by environmental stresses, high light intensity or elevated temperature can significantly increase the TPC, TFC and their corresponding antioxidant activities (Rao & Zheng, 2025; Salami et al., 2024). During early vegetative growth, when fast biomass growth is accompanied by the production of reactive oxygen species, up regulation of PAL, C4H, 4CL, CHI and F3H genes allows the accumulation of phenylpropanoids and flavonoids (Hou et al., 2023; Shamshiri et al., 2025; Shan et al., 2025). This explains why DPPH and ABTS assays followed very close to total phenolics and flavonoids: the mid-season increase in these antioxidants coincided with the highest phenolic and flavonoid concentrations, illustrating the well-known role of hydroxyl groups in neutralising free radicals through hydrogen- and electron-donating mechanisms (Ciocan et al., 2023; Šedbare, Pašakinskiene & Janulis, 2023; Tlili et al., 2023).

As the plants transition toward flowering and seed formation, carbon allocation appears to shift away from phenylpropanoid products toward terpenoid volatiles (Liu et al., 2024). For example, in rose petals, genes involved in terpene biosynthesis such as geranylgeranyl diphosphate synthase (RcGGPPS1), farnesyl diphosphate synthase (RcFPPS1/2) and various terpene synthases show distinct expression patterns (Kiani et al., 2024; Li et al., 2024a; Li et al., 2024b), with several exhibiting highest expression in late or senescent flowers and with monoterpene emissions increasing substantially after anthesis (Zhao et al., 2024; Zhu et al., 2022). Such developmental regulation implies that the mevalonate and methylerythritol phosphate pathways are upregulated late in the season, diverting carbon skeletons toward essential oil biosynthesis while phenylpropanoid flux declines (Basallo et al., 2024; Escobar-Bravo et al., 2023). The negative correlation between the content of essential oils and TFC and DPPH activity supports this metabolic trade-off. By H11, the accumulation of essential oils was at its maximum while the pool of flavonoids and phenolics was already decreasing, a similar trade-off has been observed in other aromatic species, where the rising temperature stimulates the accumulation of monoterpenes at the expense of phenolics (Bourtsoukidis et al., 2024; Mansinhos, Gonçalves & Romano, 2024; Zhu et al., 2024).

Environmental factors may help to modulate these trajectories. These factors, such as geography and climatic conditions, have been shown to strongly influence essential oil composition in *T. capitata*, particularly the relative abundance of carvacrol and its precursors (Economou et al., 2011; Tammar et al., 2019). Variations in altitude have also been reported to significantly affect both the chemical composition and biological activity of *T. capitata* essential oil (El-Jalel et al., 2018).

Exposure to high irradiance improves phenolic and flavonoid production and increases DPPH and FRAP activities in some plants (Jadidi et al., 2023; Rai et al., 2023), while high temperature in micro propagated Thymus plants increases TPC and antioxidant activities (Hashemifar et al., 2025). In the present data, the pre-flowering peak of ABTS activity may be reflecting such stimuli: increasing light intensity and temperature in midsummer may induce flavanol and phenolic acid biosynthesis, augmenting the ABTS-responsive antioxidants. After H7, declining antioxidant metrics in the face of continued essential oil accumulation indicate that these pathways are competitive for common precursors instead of acting in parallel (Ho, Yang & Lin, 2024; Ryu et al., 2024). Once reproductive structures are formed, oxidative stress and hormonal signals possibly down-regulate the phenylpropanoid genes and up-regulate the terpenoid synthases (Shan & Jin, 2025; Wang et al., 2025), in order to reallocate the resources.

This viewpoint has practical implications for harvesting management. Early harvests, when phenolic and flavonoid concentrations and antioxidant capacities are greatest, provide material with high levels of radical-scavenging phenolics that are important for nutraceutical or medicinal applications. Later harvests, which are particularly around the flowering period, maximize EOC and therefore favor production of the aromatic volatiles. Understanding the metabolic changes enables producers to optimize harvesting time to access phytochemical profiles to take advantage of the natural developmental and environmental control of these complex biosynthetic pathways.

### Developmental dynamics of essential oil composition: carvacrol and its precursors

The sequential harvests indicate a strong temporal reorganization of essential oil composition, with the relative abundances of carvacrol and the two precursors p-cymene and γ-terpinene changing with plant maturity. In the first harvest, carvacrol represented about four-fifths of the oil, whereas p-cymene and γ-terpinene hardly amounted to more than 3%. The predominance of carvacrol observed in this study is consistent with previous reports on Mediterranean populations of *T. capitata*, where this compound often represents the dominant chemotype (Miceli, Negro & Tommasi, 2006; Bakhy et al., 2013; Russo et al., 2013). By the last harvest, the relative abundances of p-cymene and γ-terpinene had reached 13.4% and 9.3% respectively, whereas carvacrol had faded to about two-thirds of the oil.

Minor monoterpene hydrocarbons such as α-thujene, β-myrcene and α-terpinene, increased several folds during the same period and the ratio of hydrocarbon monoterpenes increased from 7.5% to over 30%, while the ratio of oxygenated monoterpenes decreased from over 90% to around 68%. These shifts occurred despite a consistently high percentage of identification, suggesting that the changes are reflective of true metabolic adjustment and not analytical artefacts. These trajectories are consistent with the architecture of the monoterpenoid biosynthetic network. γ-Terpinene and p-cymene are upstream intermediates in the pathway leading to phenolic monoterpenes such as thymol and carvacrol (Alipour et al., 2025; Sonigra & Meena, 2025).

Similar biosynthetic relationships between γ-terpinene, p-cymene, and phenolic monoterpenes such as carvacrol have been previously described for *T. capitata* (Skoula & Grayer, 2005). Cytochrome P450 monooxygenases (CYP71D family) hydroxylate γ-terpinene to unstable cyclohexadienols (Ahmadi et al., 2024; Ling et al., 2023), which can be further oxidized by short-chain dehydrogenases into ketone intermediates; when this conversion is limited or bypassed, the intermediate may rearrange to p-cymene (Ahmadi et al., 2024; Shariatzadeh, Talebi & Ghorbanpour, 2025). Carvacrol is formed from γ-terpinene through sequential oxidative steps, whereas p-cymene reflects a branch point associated with incomplete oxidative processing (Shariatzadeh, Talebi & Ghorbanpour, 2025). Hence, the inverse trends observed in carvacrol *versus* p-cymene and γ-terpinene across harvests suggest a shift in metabolic flux upstream (Krause et al., 2021; Shariatzadeh, Talebi & Ghorbanpour, 2025), when hydroxylation and dehydrogenation are active early in development, downstream phenolic products dominate; as these reactions wane in later stages, precursors accumulate and the proportion of phenolic monoterpenes declines. Such reciprocity is likely representative of competition for a common precursor pool in the monoterpene biosynthetic network.

These ontogenetic patterns are modified but not overridden by environmental factors. Elevated temperatures can significantly increase monoterpene emissions in plants that contain specialized storage structures (Bourtsoukidis et al., 2024; Li et al., 2024a; Li et al., 2024b), and, in oregano, there is a positive correlation between temperature and thermal sum on the one hand and p-cymene accumulation on the other, and a negative correlation with rainfall (Abbasi, Houshmand & Ahmadi, 2024; Li et al., 2024a; Li et al., 2024b). Heat stress increases the volatilization of unsaturated hydrocarbons (*e.g.*, γ-terpinene and β-myrcene) leading to oxidation to p-cymene and other phenolics (Abbasi, Houshmand & Ahmadi, 2024; Li et al., 2024a; Li et al., 2024b). In the current results, mid-to-late harvest increases in p-cymene and γ-terpinene coincided with warmer and drier conditions, which suggests that environmental cues contribute to enhancing the shift to hydrocarbon precursors while limiting downstream oxidation (Pugliese et al., 2023; Qin et al., 2025). Nevertheless, the underlying developmental trajectory is maintained: phenolics and monoterpenes are favored at early stages; at maturity, synthesis and retention of less oxidized hydrocarbons are enhanced. This developmental reprogramming of monoterpene metabolism is like changes seen in the phenylpropanoid pathway where resources are redirected away from antioxidant phenolics in order to be used for other compounds as plants make the shift from vegetative growth to reproduction (Shan & Jin, 2025; Singh, Chhatwal & Pandey, 2024).

This explanation is also supported by principal component analysis (PCA) analyses (Fig. 4). Early samples were rich in oxygenated phenolics, and flowering stages had profiles dominated by monoterpene hydrocarbons and oxygenated sesquiterpene and a post-flowering decline followed. The directions of harvest vector and related metabolite clumps point to well-controlled biochemical transitions that accompany ontogeny and point to the fundamental importance of flowering in restructuring the volatile landscape. Overall, the temporal trends that are observed for the fourteen harvests suggest a developmentally orchestrated monoterpene biosynthesis, which is modulated by environmental factors but is largely under the control of the ontogenetic program of the plant. These findings have implications for practice. Harvests at early stages of plant development will maximize the phenolic monoterpenes such as carvacrol which are responsible for antimicrobial potency and flavor (Shariatzadeh, Talebi & Ghorbanpour, 2025), but later harvests will be richer in hydrocarbon precursors and minor terpenes which change both the aroma and biological activity. Understanding the biochemical basis of these shifts enables producers to adjust harvest schedules to target specific compositional profiles and points to the need to account for environmental conditions, particularly temperature and water availability, in interpreting the variability of essential oils.

## Conclusions

The combined evidence from sequential harvests suggests that secondary metabolism operates through coordinated reprogramming, and this can be harnessed for agricultural purposes. During the early stages of vegetative development, high phenolic and flavonoid concentrations are associated with high DPPH and ABTS activities, and the essential oil is dominated by oxygenated monoterpenes like carvacrol. Mid-season harvests will maintain moderate antioxidant capacity and still provide considerable levels of carvacrol and thymol, and as the plants come close to flowering, the levels of γ-terpinene and p-cymene will begin to accumulate and TPC will decrease. Late harvests favor monoterpene hydrocarbons (α-thujene, β-myrcene and α-terpinene increase several folds and p-cymene approaches 1/8 of the oil). These shifts represent an upstream diversion of metabolic flux from phenylpropanoid and phenolic monoterpene pathways towards hydrocarbon precursors as a trade-off enhanced under warm, dry conditions. From a production point of view, the choice of the harvest window makes it possible to adjust both bioactivity and sensory qualities. Early harvests (H1–H5) produce oils with high contents of carvacrol and phenolic compounds with high antimicrobial and antioxidant activity and could be used as nutraceuticals or preservatives. Intermediate harvests (H6–H9) give balanced profiles with moderate phenolics and a wider range of volatiles, which could contribute to a more complex aroma. Later harvests (H10–H14) favor monoterpene hydrocarbons and p-cymene, resulting in lighter and more fragrant oils, but lower TPC; the concomitant increase in α-thujene and β-myrcene may be considered an indicator of maturity and environmental stress. By knowing the developmental and environmental factors influencing these compositional trajectories, growers can strategically harvest to meet specific industrial or therapeutic targets.

##  Supplemental Information

10.7717/peerj.21519/supp-1 Supplemental Information 1 Representative GC–MS chromatogram of *Thymbra capitata* essential oil obtained at the first harvest (H1) Peak numbers correspond to the compounds listed in Table 1.

10.7717/peerj.21519/supp-2 Supplemental Information 2 Representative GC–MS chromatogram of *Thymbra capitata* essential oil obtained at the last harvest (H14) Peak numbers correspond to the compounds listed in Table 1.

10.7717/peerj.21519/supp-3 Supplemental Information 3 Dataset 1

## Additional Information and Declarations

### Data Availability

The following information was supplied regarding data availability: The raw data is available in the Supplemental File.
