# Supplementary figures and images for: Ontogenetic regulation of metabolite dynamics in Thymbra capitata: temporal reconfiguration of essential oils and antioxidant capacities across sequential harvests
Source: PeerJ. 2026 Jul 8;14:e21519. doi: 10.7717/peerj.21519 (PMC13355610; doi:10.7717/peerj.21519)

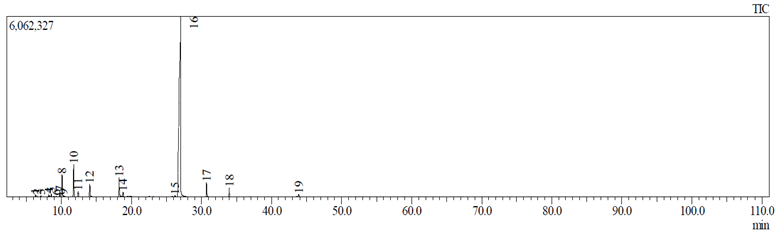

Supplement: Supplemental Information 1 — Peak numbers correspond to the compounds listed in Table 1. [file peerj-14-21519-s001.png]

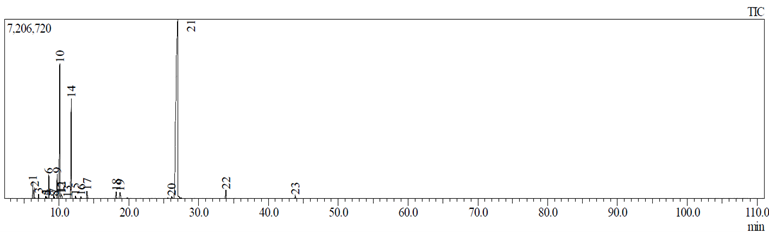

Supplement: Supplemental Information 2 — Peak numbers correspond to the compounds listed in Table 1. [file peerj-14-21519-s002.png]
